# A Hybrid CPU-GPU Accelerated Framework for Fast Mapping of High-Resolution Human Brain Connectome

**DOI:** 10.1371/journal.pone.0062789

**Published:** 2013-05-10

**Authors:** Yu Wang, Haixiao Du, Mingrui Xia, Ling Ren, Mo Xu, Teng Xie, Gaolang Gong, Ningyi Xu, Huazhong Yang, Yong He

**Affiliations:** 1 Department of Electronic Engineering, Tsinghua University, Beijing, China; 2 State Key Laboratory of Cognitive Neuroscience and Learning, Beijing Normal University, Beijing, China; 3 Microsoft Research Asia, Beijing, China; Cuban Neuroscience Center, Cuba

## Abstract

Recently, a combination of non-invasive neuroimaging techniques and graph theoretical approaches has provided a unique opportunity for understanding the patterns of the structural and functional connectivity of the human brain (referred to as the human brain connectome). Currently, there is a very large amount of brain imaging data that have been collected, and there are very high requirements for the computational capabilities that are used in high-resolution connectome research. In this paper, we propose a hybrid CPU-GPU framework to accelerate the computation of the human brain connectome. We applied this framework to a publicly available resting-state functional MRI dataset from 197 participants. For each subject, we first computed Pearson’s Correlation coefficient between any pairs of the time series of gray-matter voxels, and then we constructed unweighted undirected brain networks with 58 k nodes and a sparsity range from 0.02% to 0.17%. Next, graphic properties of the functional brain networks were quantified, analyzed and compared with those of 15 corresponding random networks. With our proposed accelerating framework, the above process for each network cost 80∼150 minutes, depending on the network sparsity. Further analyses revealed that high-resolution functional brain networks have efficient small-world properties, significant modular structure, a power law degree distribution and highly connected nodes in the medial frontal and parietal cortical regions. These results are largely compatible with previous human brain network studies. Taken together, our proposed framework can substantially enhance the applicability and efficacy of high-resolution (voxel-based) brain network analysis, and have the potential to accelerate the mapping of the human brain connectome in normal and disease states.

## Introduction

In recent years, increasing attention has been paid to the structural and functional connectivity patterns of the human brain, i.e., the human connectome [1]. An important and promising way to study the human connectome is to combine non-invasive neuroimaging techniques (e.g., structural MRI, functional MRI, and diffusion MRI) and graph theoretical approaches (for reviews, see [2]–[5]). Under this framework, the brain is modeled as a complex network that contains a large quantity of nodes and connections. The brain nodes are usually defined by imaging voxels or regions of interest (ROIs); the brain edges are defined by measuring the structural or functional association between the nodes based on neuroimaging data. Once the brain networks are constructed, various graph-based metrics can be used to measure the underlying topological properties of the networks, such as small-worldness, network efficiency, modules, and hubs. To date, graph theoretical analysis of neuroimaging data has been widely used to study the topological architecture of the human brain connectome in normal adults [6]–[13], during development [14]–[18] and throughout the aging process[6], [19]–[22]. Moreover, these methods have also been used to reveal topological alterations of neurological and psychiatric diseases such as Alzheimer’s disease [23]–[25], schizophrenia [26]–[28], and depression [29].

In spite of these advances, there are many unresolved issues in the human brain connectome field. One such issue is the required high computational capability that results from the following factors. 1) Network size. The size of the brain networks keeps increasing with the spatial resolution of the imaging techniques. This increase leads to an almost unacceptable running time for the construction and analysis of voxel-based brain networks (the order of 10^4^ nodes) on a single CPU because the running time of most graph theoretic algorithms increases super-linearly with the network size. 2) Datasets. The increasing number and size of datasets are generated in the community, for example, from the 1000 Functional Connectomes Project (www.nitrc.org/projects/fcon_1000/). The increase in the number of subjects is important for the statistical power of the analysis results but leads to an increase in the running time. 3) Repeated experiments. To investigate the effects of the empirical parameters (e.g., network thresholding) and the preprocessing steps (e.g., global signal removal and head motion) on the network analysis results, brain network construction and analysis processes are usually performed many times. 4) Random network calculation. Some topological parameters (e.g., small-world and modular analysis) involve the characterization of many matched random networks, which requires extra computation. All of these factors lead to the computational intractability of high-resolution brain network analysis.

Given the limitations of the available computational power, several compromises have been widely used in human connectome studies. For example, a number of researchers have employed a small number of ROIs to define the network nodes [8]–[30] or have down-sampled the imaging data into a coarse level [7], [31], [32]. Obviously, ROI-based networks are quite sensitive to the choice of brain parcellation, which raises an extra issue for the ROI definition [28], [33]. Moreover, this type of low-resolution networks also leads to the loss of some important connectivity information [34], especially for regions that contain multiple sub-divisions (e.g., precuneus [35]). Notably, voxel-based networks have the highest resolution and have naturally-defined brain nodes (i.e., voxels) and, therefore, can overcome the above limitations of ROI-based networks. However, voxel-based network analysis requires a very large computing capability because of having a very large network size (approximately 58 K). Thus far, very few studies have used voxel-level brain networks; these studies mainly focused on graph theoretical metrics that had a lower computational complexity (e.g., nodal degree) [7], [36], [37]. The graph theoretic properties that had high computational loads (e.g., small-worldness, modular structure and network efficiency) are rarely studied in high-resolution voxel-based brain networks.

Recent advances in Graphics Processing Units (GPUs) provide a promising solution to the above-mentioned difficulties. GPUs were originally intended for the fast manipulation and display of graphics. Graphics processing has three key characteristics [38]: a large amount of parallelism, little data reuse, and a high computation-to-memory access ratio. Therefore, GPUs are designed with a many-core architecture that favors massively data-parallel computing but that has a small cache and simple flow control. At the turn of this century, researchers proposed that GPUs could also be applied to a broad range of general-purpose applications other than graphics processing. Over the past decade, GPUs have proven to be useful in many general-purpose computing fields, such as scientific computation, video coding and encoding and pattern recognition. Many graph theoretical algorithms can benefit from GPU acceleration because of the high degree of parallelism in these algorithms. Nevertheless, these facts do not imply that GPUs always outperform CPUs in network analysis. For example, a GPU is less efficient than a CPU when calculating the clustering coefficients and the characteristic path length for some sparse graphs. Therefore, it is desirable to combine CPUs and GPUs to obtain better performance.

In this study, we proposed a heterogeneous platform comprising multi-core CPUs and GPUs to accelerate the graph theoretical algorithms that are used in high-resolution (voxel-based) functional brain network analysis. The acceleration framework mainly included the construction of voxel-based brain networks and graph-theory analysis. To test our platform, we obtained a set of high-resolution functional brain networks with ∼58 K nodes and a sparsity range from 0.023% to 0.151%. We then analyzed a variety of global (e.g., clustering coefficient, shortest path length, small-worldness, efficiency and modules) and regional (e.g., nodal degree) network characteristics and compared them with the average metrics of 15 random networks. The entire process for one network was completed in 80∼150 minutes, depending on the network sparsity.

### GPU Programming Model and Architecture

**Figure 1:**
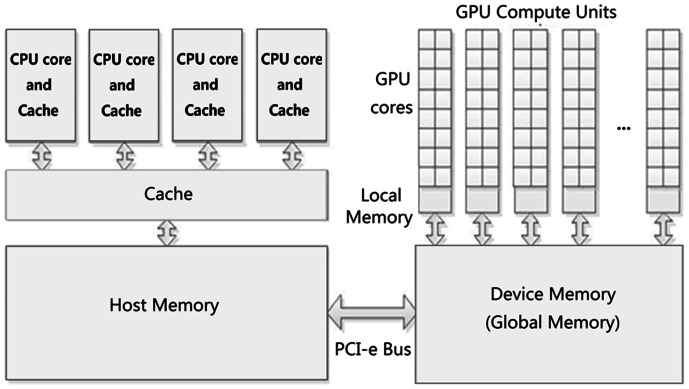
CPU-GPU hybrid hardware platform. The communication between the host (CPU) and device (GPU) is through a PCI-e bus.

In this section, we briefly introduce the CUDA programming model for GPUs and the hybrid CPU-GPU hardware platform (illustrated in Fig. 1). When introducing the GPU architecture, we take NVIDIA Geforce GTX 580 GPU as an example.

CUDA (Computing Unified Device Architecture) is a parallel computing platform and programming model created by NVIDIA; it leverages the parallel compute engine in NVIDIA GPUs to solve many complex computational problems in a more efficient way than on a CPU (www.nvidia.com/object/cuda_home_new.html). A typical GPU program includes three main steps: copying data from CPU memory to GPU memory, GPU kernel executions, and reading data back from GPU memory to CPU memory. A kernel, representing a function running on a GPU, is executed by a number of threads. The thread hierarchy - threads are grouped into blocks, and blocks are organized into a grid - enables efficient cooperation between threads and a hierarchical mechanism of memory access. For example, threads within the same block can share data through shared memory with low latency and can synchronize their execution, while all of the threads have their own register, and share the *global memory*. Threads within a thread-block can be identified using thread indices (called *threadIdx*).

A GPU consists of an array of *Streaming Multiprocessors* (SMs,16 SMs for GTX 580), each executing one or more block(s) concurrently. In a *Streaming Multiprocessor*, there are a number of *processing units* (32 processing units for GTX 580), each executing several threads concurrently. The *Streaming Multiprocessors* schedule threads in an SIMD-manner (Single Instruction Multiple Data). An on-chip *local memory* or *shared memory*, enabling local data shares, and a register file are available on each of the *Streaming Multiprocessors*. Using registers and local memory or shared memory is typically an order of magnitude faster than accessing global memory, which is one order of magnitude faster again than accessing CPU memory. (CUDA Programming Guide from http://developer.nvidia.com/).

With far more processing cores than CPUs, GPUs possess much higher computational capability and also higher memory bandwidth. However, even for an algorithm with considerable parallelism, it is not always easy to achieve the full potential of a GPU. A series of modifications must be made to adapt the algorithms to the specific GPU architecture. Usually, two considerations are crucial to the performance: 1) a sufficient number of concurrent threads must be invoked to fully utilize the numerous cores on the GPU; 2) the accesses must be minimized to slow the memory and to use fast memory when possible.

## Materials and Methods

### Data Acquisition and Preprocessing

The dataset was downloaded from the 1000 Functional Connectomes Project (www.nitrc.org/projects/fcon_1000/), which is a worldwide multi-site project with fMRI data sharing for the imaging community. The dataset we used was from Dr. Yu-Feng Zang, Beijing. The resting-state images were acquired from 198 healthy right-handed volunteers, comprising 76 males and 122 females, age 21.2±3.3 years (ranging from 18 to 26 years old). We excluded one subject’s data because of an orienting error during scanning. Each participant signed a written informed consent before the scanning. The study was approved by the Institutional Review Board of the Beijing Normal University Imaging Center for Brain Research.

The acquisition was performed on a *Siemens 3* *T* scanner. For each participant, functional images were scanned using the following parameters: time points = 225, repetition time = 2000 *ms*, echo time = 30 *ms*, in-plane resolution = 3.125 *mm*×3.125 *mm*, slice thickness = 3 *mm*, number of slices = 33, section gap = 0.6 *mm*, flip angle = 90°, and field of view = 200 *mm*×200 *mm*. The participants were instructed to close their eyes and stay awake during the scanning.

All of the image preprocessing was conducted using DPARSF [39] and SPM5 (www.fil.ion.ucl.ac.uk/spm/). The first 10 volumes on each participant were removed because of signal equilibrium and to allow the participants’ adaptation to the scanning noise. The following preprocessing steps included slice timing, realignment, normalization into standard MNI space with EPI as a template and resampled to voxel size 3 *mm*×3 *mm*×3 *mm*, detrend, and a band-pass filtering from 0.01 to 0.08 Hz. Furthermore, several frequently used noise reduction strategies were utilized, including the regression of white matter (WM), cerebrospinal fluid (CSF), global mean signal time courses, and head-motion profiles. To restrict subsequent functional analysis within gray matter tissues, we generated a gray matter mask as follows. First, we resampled the gray matter tissue probability map provided by SPM5 into 3 *mm*×3 *mm*×3 *mm* resolution. Then we binarized the resampled probability map by a threshold of 0.2, which resulted in a gray matter mask of 58523 voxels.

### The Proposed Hybrid CPU-GPU Framework

**Figure 2:**
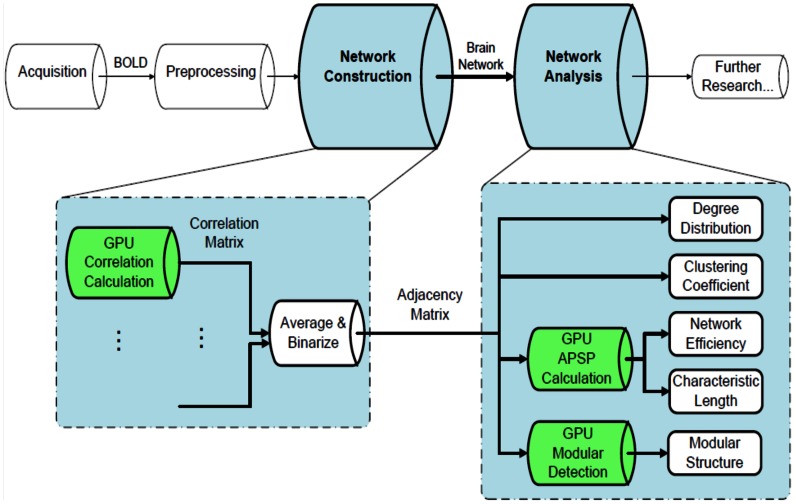
The work flow of the proposed framework. There are four steps: data acquisition, image preprocessing, network construction and network analysis. We accelerate the latter two steps (shown in detail in the two blocks at the bottom). In network construction, correlation matrices for each subject are obtained by calculating Pearson’s Correlation coefficients. These correlation matrices are then “binarized” into adjacency matrices. In network analysis, several characteristics are calculated. Procedures denoted in green (i.e., Pearson’s Correlation, Partition and APSP) are accelerated with the GPU. Other procedures are implemented with multi-threads on a multi-core CPU.

Our proposed CPU-GPU framework comprises two main parts: network construction and network analysis (for the work flow, see Fig. 2). The inputs of our system were the pre-processed fMRI data in NIfTI format of each subject. In the network construction, the first step was to calculate Pearson’s Correlation [40] coefficients for every pair of voxels, to obtain a correlation matrix for each subject. These correlation matrices were then “binarized” (details presented in the section on Network construction) into Boolean adjacency matrices. In the network analysis, we computed several graphic characteristics, including the nodal degree, the clustering coefficient, the characteristic path length, the global efficiency and the modular structure. The definition of these network characteristics can be found in many studies [33], [41]. Pearson’s Correlation, modular detection and APSP were accelerated with GPUs, and the other processes were accelerated with multi-core CPUs (Fig. 2). For the CPU programs, we used an Intel(R) Core(TM) i7-3770 quad-core CPU @ 3.4 GHz with 32 GB RAM. For the GPU programs, we used the NVIDIA Geforce GTX 580, with the CUDA Toolkit v4.2 and the GPU computing SDK v4.2 (http://developer.nvidia.com/cuda/cuda-downloads). The operating system was Windows 7.

### Network Construction

#### 1) Pearson’s Correlation

For the voxel pair (*v_i_*, *v*_j_), Pearson’s correlation [40] between the time series of the pair is defined as follows:
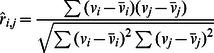
where column vector *v_i_* denoted the time series of voxel *i*, was the average of the series of voxel *i*, and all of the Σ symbols denoted 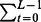, i.e., summing along the whole time series (*L* was the length of the time series). First, we normalized the fMRI time series, 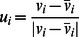, **U** = (*u_1_*, *u_2_*, …, *u_N_*) was the aggregate of the normalized time series. The whole pair-wise correlation matrix for the *N* voxels was computed by a matrix multiplication **R** = **U** ^T^ **U**. Matrix multiplication is very efficient on a GPU, e.g., 645 Gflop/s (Giga floating-point operations per second) on the NVIDIA Fermi GPUs (e.g., the Geforce GTX 580) [42]. Because the sizes of the voxel-based brain networks were very large, the correlation matrix **R** sometimes exceeded the GPU memory. To address this problem, we divided the matrix **U** into *m* blocks, using a preset block size (e.g., 3072×3072) **U = (U_1_, U_2_, …,U_m_)**, which implies that



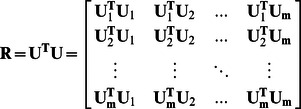



In this way, our platform could handle networks of any size and was not limited by the graphic memory. One block is calculated each time. Once a block was transferred back to the CPU, the GPU started calculating the next block. Considering the symmetry of the correlation matrix, only the upper half of the matrix must be calculated.

#### 2) Adjacency matrix construction

The aim of this study is to illustrate the contribution of many-core computing systems (GPUs) to voxel-based brain network analysis. Instead of investigating the discrepancy between subjects, we focused on the brain network properties that people have in common. Thus, we averaged the correlation matrices of the 197 subjects. Then, we used a set of thresholds 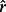 to binarize the mean correlation matrix to obtain a set of sparse, unweighted networks, or adjacency matrices. Specifically,
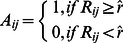
where *A* was the adjacency matrix and *R* was the mean correlation matrix. The range of thresholds controlled the sparsity of the network *S* to satisfy *S_1_< S<S_2_*. The lower bound *S*_1_ was determined by the average degree. To maintain estimable small world properties of the network, the average degree *k* should satisfy *k*>log(*N*), where *N* was the number of voxels in the network, and in this case, *N* is 58523. Therefore, *S*_1_ could be calculated as



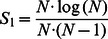



The upper bound *S*_2_ was determined by the threshold 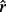, which corresponds to the significance level of 0.05 (Bonferroni-corrected). From the calculation, *S*_1_ and *S*_2_ were 0.019% and 0.174%, respectively, and the corresponding threshold 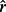 ranged from 0.43 to 0.73. For convenience, we chose the following six thresholds, 0.45, 0.5, 0.55, 0.6, 0.65, and 0.7, and thus obtained unweighted networks with corresponding sparsities of 0.151%, 0.098%, 0.067%, 0.047%, 0.033%, and 0.023%.

### Network Analysis

Here, we calculated the nodal degree, and the clustering coefficient (*Cp*), the characteristic path length (*Lp*) for the unweighted brain networks. These characteristics reveal interesting properties of functional brain networks such as the small-world property and the degree distribution. Notably, *Lp* relies on the results of All-Pairs Shortest Path (APSP), one of the most time-consuming steps in voxel-based brain network analysis. We specifically introduce the acceleration for APSP into the section on Network Analysis, in which different APSP algorithms were adopted for networks of different sizes and sparsities. Another time-consuming step in brain network analysis is modular detection. Our GPU acceleration for modular detection is presented in the section on Network Analysis.

#### 1) All-pairs shortest paths calculation

There are two main classes of APSP algorithms. One class is Johnson’s algorithm [43], which is based on single-source shortest path algorithms, such as the Dijkstra algorithm [44] and the Bellman-Ford algorithm [45]. When applied to unweighted graphs, Johnson’s algorithm reduces to Breadth-First Search (BFS). Johnson’s algorithm and BFS are efficient with sparse graphs but perform poorly with dense graphs. The other class is the Floyd-Warshall (FW) algorithm [46], [47], which, unlike Johnson’s algorithm, has *O*(*N* ^3^) time complexity (which is irrelevant to the network sparsity) and favors dense networks. The blocked FW algorithm [48] is an improved version of the basic FW algorithm and is more suitable for parallelization. Considering that the brain networks are usually modeled at different sparsities, both of these algorithms are useful. We provide both of the algorithms in our toolbox to allow users to be able to make an optimal choice according to the network sparsity that they have. In this study, we implemented both the multi-thread BFS on multi-core CPUs and the blocked FW algorithm on GPUs, and we set aside the study of the GPU acceleration for BFS for future work. The implementation of the multi-thread BFS on CPUs was straightforward. All of the threads traversed across all of the graph vertices as source points. Each thread was responsible for its proportion of source nodes and found the adjacent and unvisited vertices iteratively. It is worth noting that sorting the vertices according to their degree will benefit the load balance between different threads.

**Figure 3:**
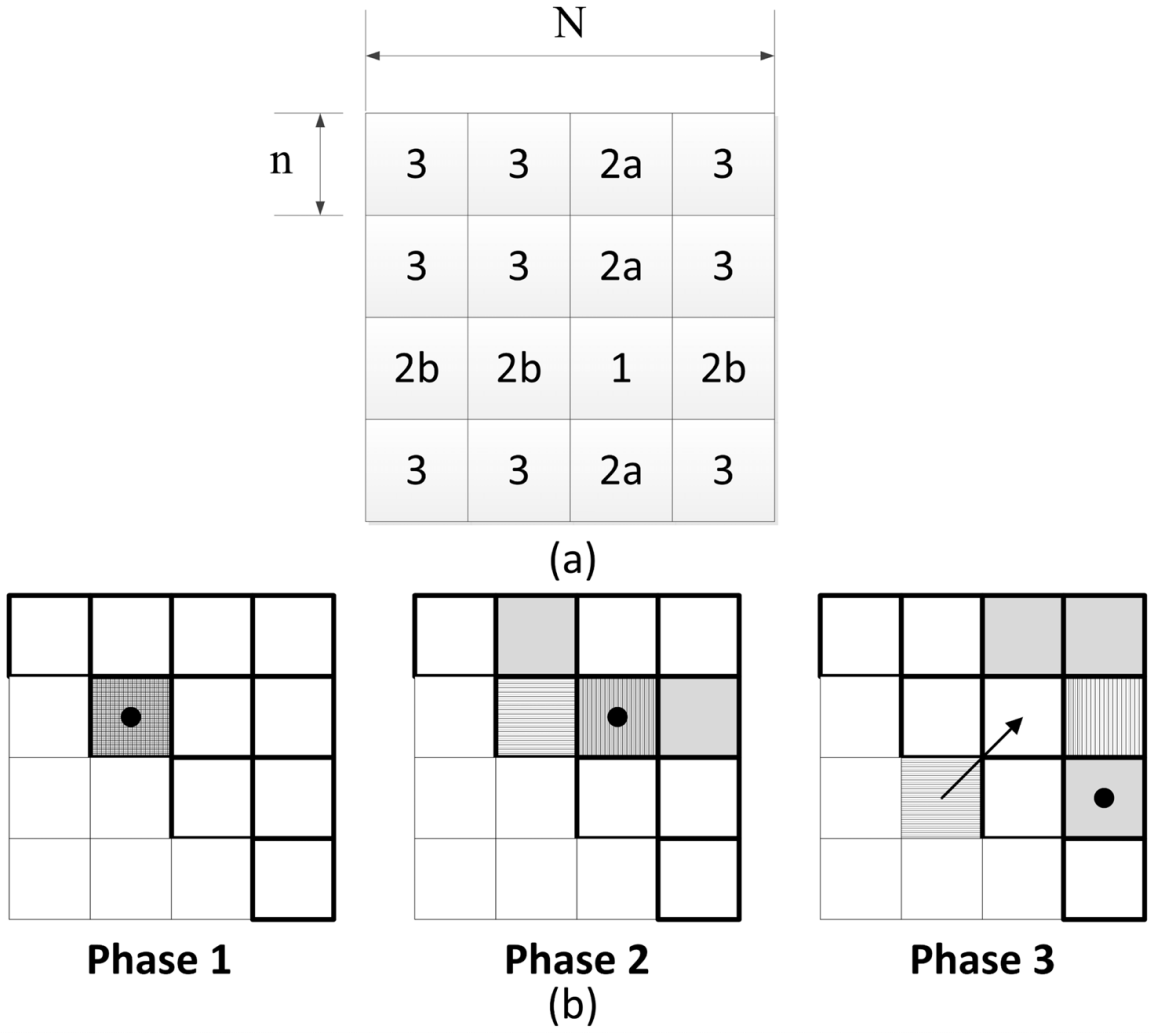
The process of the blocked Floyd-Warshall algorithm in a round. (a) Illustration of which phase each block belongs to. In a certain round, Phase 1 is a primary block. Blocks that share the same row or the same column with the primary blocks are in Phase 2. All of the other blocks are in Phase 3. (b) Updating the dotted block requires two source blocks: 1) the block in the same column as itself and in the same row as the primary block, denoted with vertical lines, and 2) the block in the same row with itself and in the same column with the primary block, denoted with horizontal lines. Because we store only the upper block triangular matrix, some source blocks in Phase 3 do not exist, in which case we transpose the corresponding existing blocks to serve as the source blocks.

In the blocked FW algorithm, the whole adjacency matrix was first converted to an *N*×*N* cost matrix C, where *N* was the number of voxels. C*_ij_* was the distance from voxel *i* to voxel *j*, or ∞ if there was no such path. Then, the cost matrix was divided into *r n*×*n* sub-blocks, where 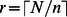. The outer loop iterated over the *r* primary blocks (the blocks along the diagonal of the matrix). Each round was divided into three sequential phases. Fig. 3(a) shows to which phase each block belonged. Each block was updated in a similar fashion as in the basic FW algorithm [46], [47], which is specifically the following:
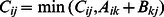
where element (*k*, *k*) was in the primary block. Updating a block required two source blocks, as shown in Fig. 3(b), (1) the block in the same column as itself and in the same row as the primary block, denoted with vertical lines, and (2) the block in the same row as itself and in the same column as the primary block, denoted with horizontal lines. The basic operations in the blocked FW algorithm is similar to matrix multiplication [49] or is referred to as generalized matrix multiplication. Following the ideas of generalized matrix multiplication (GEMM), the blocked FW algorithm could also be implemented efficiently on GPUs. Matsumoto implemented the APSP using blocked FW algorithm on GPUs [50].

We further proposed two optimizations. First, Phase 1 in the blocked FW algorithm was the basic FW algorithm. However, to represent all of the computation with generalized matrix multiplication, another algorithm with *O*(*n* ^3^log_2_ *n*) time complexity was adopted in [50]. Actually, it is more efficient to apply the blocked FW algorithm again for Phase 1. In this way, we did not bring in any extra computation. Second, the brain has often been modeled as a symmetric network. In this case, only the upper half of the cost matrix must be updated, which means that only *r*(*r+*1)*/*2 blocks must be updated in each round. However, if only the upper half matrix was maintained, then some source blocks did not exist (see Fig. 3(b)), and we needed to transpose the blocks at the symmetric location.

#### 2) Modular detection

There are currently several modular detection methods that are applicable to un-weighted networks. A random-walk-based method was introduced in [51], and was used to identify modules in voxel-level brain networks in [52]. A greedy algorithm was presented in [53], and was used to uncover the modular structure in region-based brain networks in [12]. The algorithm we chose for modular detection was the eigenvector-based spectral partition method [54]. This algorithm was more precise compared to the others but, at the same time, involved much more computation than the above approximate algorithms. Our GPU implementation greatly accelerated this algorithm and made it applicable to very large graphs. The idea of modular detection is to find groups of nodes that have many inner-group connections and relatively few inter-group connections. A benefit function *Q* was introduced to judge the network’s modularity, which was defined as follows:
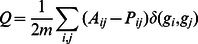
where *A_ij_* was an element of the binary adjacency matrix; *P_ij_* was the probability for an edge to fall between every pair of vertices *i, j*; *g_i_* indicated the community to which vertex *i* belongs; *δ*(*g_i_*, *g_j_*) was 1 if *g_i_* = *g_i_* and 0 otherwise; and *m* was the number of edges in the network. *P_ij_* could be defined as *P_ij_* = *k_i_ k_j/_*2*m* where *k_i_* was the degree of node *i*. The problem then became finding the best division that maximized *Q*. Newman has proven [54] that the best division could be obtained from the eigenvector that corresponds to the most positive eigenvalue of **B**, a real symmetric matrix called the modularity matrix, with its elements *B_ij_* = *A_ij_* – *P_ij_*. Thus,



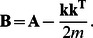



The power method [55] was used to obtain the most positive eigenvalue of **B** and its corresponding eigenvector. In the power method, we started with a random initial **x_0_** and iteratively multiplied it with **B** to obtain
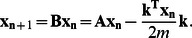



Therefore, the core operations of Newman’s spectral partition method were some basic matrix and vector operations, such as sparse matrix multiplication and the vector dot product. These operations had a high parallelism and were very suitable for GPU implementation. We constructed a GPU runtime library of these linear algebra algorithms to accelerate the power method on the GPUs.

**Figure 4:**
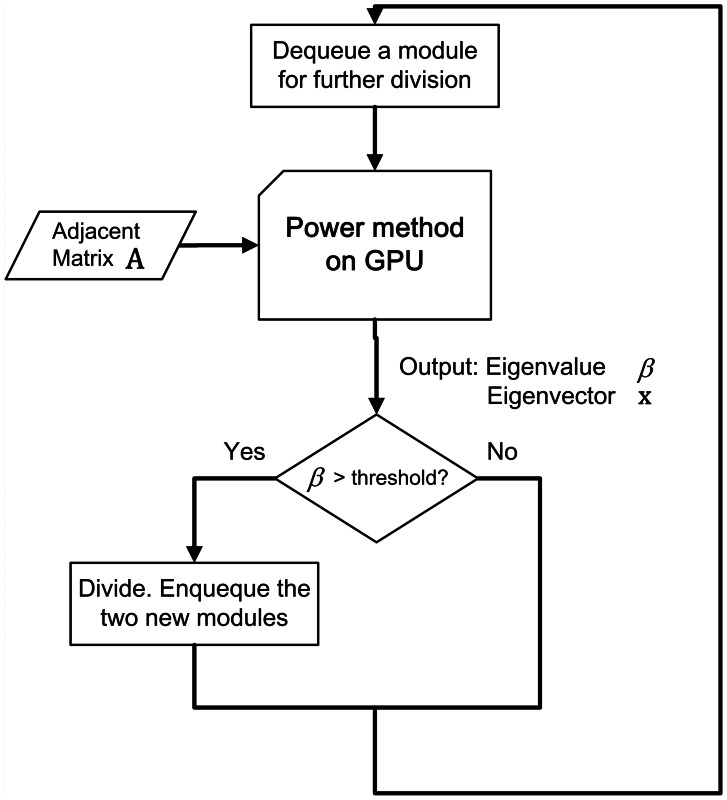
Flow Chart of our GPU implementation of Newman’s Modular Detection Algorithm. Each time, a module is dequeued. If *_β_* >0, the module is divided and the two new modules are enqueued. Repeat the process until the queue becomes empty.

After the eigenvector computation, the networks were divided into two groups according to the signs of each element in the eigenvector. Brain networks, however, are unlikely to have only two communities. A modified algorithm for handling the multiple divisions is also described in [54]. We set up a division queue on the CPU for the task scheduling. The whole partition flow is illustrated in Fig. 4. First, the whole network was enqueued as a single module. Then, we iteratively performed the following steps: dequeued a module; used the power method on the GPU to obtain the best division in this module, i.e., the eigenvector **x** of the most positive eigenvalue *β*; if *β* >0, which meant that this division would increase the benefit function *Q*, we divided the current module and enqueued the two new modules and did nothing if *β* <0. The entire division process finished when the queue became empty. A detailed description of our workflow, GPU implementation for sparse matrix vector multiplication and power method as well as more detailed optimization can be found in our previous work [56].

## Results

### Speedup and Performance

Our GPU-based Pearson’s correlation, Floyd-Warshall algorithm and modular detection on networks with ∼58 K nodes and ∼1.7 G possible edges were much more efficient than the traditional single-thread CPU implementation (Table 1). Network construction using Pearson’s correlation on GPUs was finished in 3 seconds, which was 116 times faster than the traditional single-thread CPU implementation.

**Table 1:** A comparison of the time consumed between our hybrid framework and a single-thread CPU implementation.

Sparsity	0.023%	0.033%	0.047%		0.067%		0.098%		0.151%
**Pearson’s Correlation (for each subject)**									
GPU	2.88								
single-thread CPU	335								
Speedup	116								
**All-Pairs-Shortest-Path**									
GPU BFW	1018.43	1019.57	1018.46	1018.75		1018.60		1021.36	
1-thread CPU BFW	>2.30×10^5^								
Speedup	>200								
8-thread BFS	43.10	51.52	60.97	75.06		97.34		130.14	
1-thread CPU BFS	193.18	225.66	274.90	350.44		441.78		572.53	
Speedup	4.48	4.38	4.51	4.67		4.54		4.40	
**Modular Detection**									
GPU	220.45	68.59	181.45	193.80		189.73		218.67	
1-thread CPU	373.56	149.91	419.15	457.06		448.97		568.29	
Speedup	1.70	2.19	2.31	2.36		2.37		2.60	

Note: BFW, Blocked Floyd-Warshall; BFS, Breadth First Search;The running time is given in seconds.

**Figure 5:**
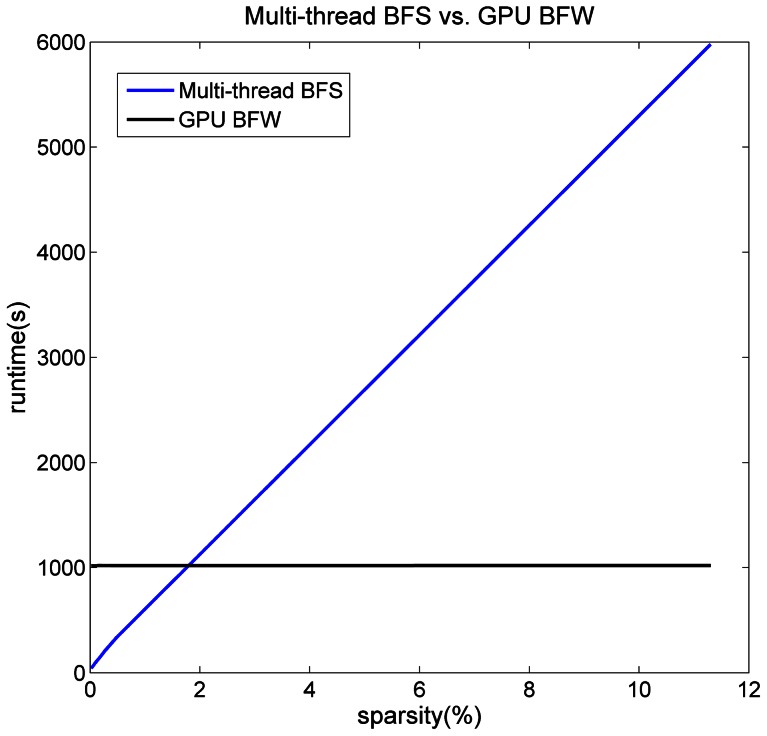
Performance of different APSP algorithms on different platforms. The blue line corresponds to the 8-thread BFS algorithm on the quad-core CPU, which is suitable for sparse networks. Its running time is proportional to the sparsity. The black line corresponds to the blocked FW algorithm on the GPU, which is suitable for dense networks. Its running time is irrelevant to the sparsity. The intersection point of the two lines is approximately where the sparsity equals 2%. This criterion is guidance for making the choice.

The blocked Floyd-Warshall algorithm was employed to calculate APSP and *Lp* on the GPUs. This procedure cost approximately 1019 seconds in all of the six sparsities, which was at least 200 times faster than the single-thread CPU implementation. We also tested another algorithm for APSP calculations called the BFS algorithm, on a quad-core CPU with 8 threads, which cost 43.1, 51.5, 61.0, 75.1, 97.3 and 130.1 seconds for networks with sparsities of 0.023%, 0.033%, 0.047%, 0.067%, 0.098% and 0.151%, respectively. Furthermore, the time complexity of this method was O(M), where M was the number of edges in a network, because the algorithm must access almost every edge in the network, and thus, with the same number of nodes, the sparsity of a network impacts the running time in a linear way. On the other hand, the running time of the blocked FW algorithm remained unchanged under different sparsities (Fig. 5). The fitted curve of the running time verses the network sparsity has suggested that for networks (on a scale of ∼58 K nodes) with a sparsity lower than 2%, 8-thread BFS on the quad-core CPU was faster; otherwise, the GPU for the blocked FW was faster.

Modular detection on the GPUs cost 220.45, 68.59, 181.45, 193.80, 189.73 and 218.67 seconds for networks with sparsities of 0.023%, 0.033%, 0.047%, 0.067%, 0.098% and 0.151%, respectively. The speedup over a single-thread implementation on the CPU ranged from 1.6 to 2.6 times, mainly depending on the sparsity of the networks. Usually, the speedup was greater on the denser networks because of the larger advantage of the GPU against the CPU when multiplying vectors with a denser matrix (sparse matrix vector multiplication is the most frequent operation in Newman’s modular detection algorithm).

Because of the different advantages of the CPU and the GPU in different procedures, we proposed a CPU/GPU hybrid framework for brain network analysis (Table 2). Consider the network with a 0.151% sparsity, for example. First, the Pearson’s correlation was performed on the GPU and cost 2.9 seconds to obtain a correlation matrix on one individual. Then, a group matrix was calculated by averaging the correlation matrix across all of the subjects on the CPU. Next, 7.5 seconds were spent for the calculation of *Cp* on the quad-core CPU. Additionally, *Lp* was calculated on the quad-core CPU using 8-thread BFS, which cost 130.14 seconds because of the low network sparsity. (The GPU FW algorithm would be employed instead if the sparsities of the network were greater than 2%; See Fig. 5.) Finally, modular detection was performed on the GPU, which cost 218.67 seconds. In the calculation of *Cp*, *Lp* and modular detection for random networks, Maslov random networks [57] were generated by a single thread on the CPU (generation only, calculation excluded), which cost approximately 18 seconds to generate one random network. For the chosen hardware and the running time of each procedure under other sparsities, see Table 2 for details.

**Table 2:** Running time for computing each network metric on the CPU, GPU and CPU-GPU hybrid.

Network Metrics	Hardware	Sparsity						
		0.023%	0.033%	0.047%	0.067%	0.098%		0.151%
Correlation	GPU	∼2.88 (*197 subjects)						
Degree	CPU	<10^−3^	<10^−3^	<10^−3^	<10^−3^	<10^−3^	<10^−3^	
*Cp*	CPU	6.3	6.2	6.3	6.3	6.7	7.5	
*Lp*	Hybrid	43.10	51.52	60.97	75.06	97.34	130.14	
Modular detection	GPU	220.45	68.59	181.45	193.80	189.73	218.67	
Maslov rewiring	CPU	204.9	211.8	214.9	224.7	238.9	270.0	

Note: The running time is given in seconds. The running time of the Maslov rewiring represents the total time for constructing 15 random networks.

Our framework has greatly accelerated the process of brain network analysis. For a network with ∼58 K nodes and ∼1.7 G possible edges, the total running time for the network construction and characteristics analysis including small-worldness, degree and modularity (comparing to 15 degree-matched random networks when needed) were approximately 80∼150 minutes, which corresponded to sparsities from 0.023% to 0.151%. In contrast, the same process executed by a single thread would cost approximately 20 to 25 hours on a CPU. Our hybrid framework saved a tremendous amount of time and energy for such a graph theoretical network analysis method.

### Network Characteristics

#### 1) Small-world properties

We calculated the clustering coefficient and characteristic path length of the voxel-based functional brain networks under all six sparsities (the geometric average of *Cp* was 0.41, ranging from 0.27 to 0.50, and the geometric average of *Lp* was 13.47, ranging from 22.3 to 7.6) and the corresponding nodes, mean degree and degree distribution matched the random networks (the geometric average *Lp_rand_* was 3.33, ranging from 4.29 to 2.73). The clustering coefficients of the functional brain networks were much higher than those of the random networks (the geometric average of *γ* was 220.5, ranging from 580 to 67). On the other hand, although the characteristic path lengths of the functional brain networks were revealed to be higher than those of the random networks in the extremely sparse situation, *Lp* was approximately equal in both the functional brain networks and the random networks across most of the sparsities (the geometric average of *λ* was 4.04, ranging from 5.21 to 2.79). In general, our results from the network analysis suggested that voxel-based functional brain networks exhibited significant small-world properties when compared to those random networks that had the same number of nodes, mean degree and degree distribution (the averaged *σ* was 54.5, ranging from 111.3 to 24.0; see Table 3).

**Table 3:** Small-world properties of brain networks, and a comparison with random networks.

Sparsity (*S*)	0.023	0.033	0.047	0.067	0.098	0.151
Clustering Coefficient						
*Cp*	0.27	0.37	0.43	0.46	0.48	0.50
*Cp_rand_*± std	4.72×10^−4^±2.0×10^−5^	8.07×10^−4^±1.3×10^−5^	1.41×10^−3^±2.1×10^−5^	2.44×10^−4^±1.6×10^−5^	4.28×10^−3^±1.6×10^−5^	7.47×10^−3^±1.5×10^−5^
*γ*	580	458	305	189	112	67
Characteristic Path Length						
*Lp*	22.32	18.77	15.28	12.29	9.94	7.64
*Lp_rand_*± std	4.29±× 10^−4^	3.80±2.3×10^−4^	3.42±1.5×10^−4^	3.10±2.2×10^−4^	2.90±1.6×10^−4^	2.74±5.8×10^−4^
*λ*	5.21	4.94	4.47	3.96	3.42	2.79
Small-worldness						
*σ*	111.3	92.7	68.2	47.7	32.7	24.0

Note: *Cp*, the average clustering coefficient of all of the nodes in the brain network; *Cp_rand_*, the average clustering coefficient of all of the nodes in the Malslov rewiring random networks; *γ = Cp/Cp_rand_*, *Lp*, the characteristic path length of the brain network; *Lp_rand_*, the characteristic path length of the Maslov random networks; *λ = Lp/Lp_rand_*; and *σ* = *γ/λ*. All of the results of the random networks are the average of 15 random networks, with the standard deviations given in brackets.

#### 2) Modularity

**Figure 6:**
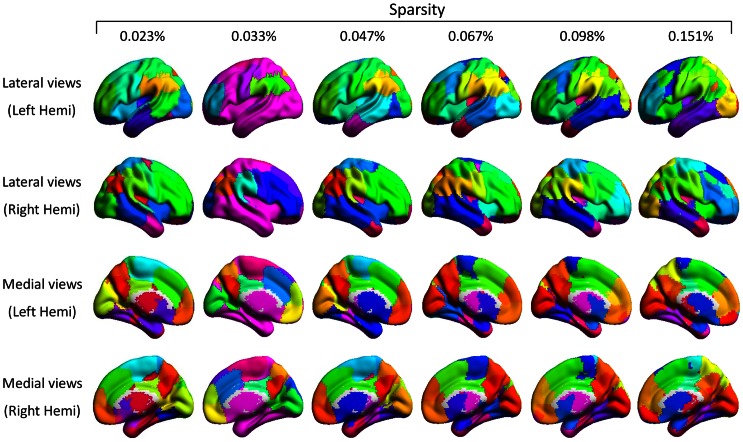
The modular structure of brain networks under six sparsities. The brain is divided into 48, 22, 46, 46, 46, and 52 modules with Q = 0.88, 0.72, 0.86, 0.84, 0.81, and 0.74 in real networks and with a sparsity of 0.023%, 0.033%, 0.047%, 0.067%, 0.098%, and 0.151%, respectively. This figure was visualized with the BrainNet Viewer (http://www.nitrc.org/projects/bnv/).

The modularity coefficients of the real brain network were relatively stable for different sparsities, while those of the random networks decreased monotonically when the sparsity threshold increased. Furthermore, the real brain network showed significant non-random modular structure over each sparsity threshold when compared to random networks. The mean modularity coefficient *Q* was 0.81±0.064 in real brain networks and the corresponding mean Z score was 13.72±1.06. These results demonstrated that there was significant non-random modular organization of the voxel-based resting-state fMRI functional human brain networks. We further visualized the modular structure onto smoothed brain surfaces for every sparsity threshold (Fig. 6). A total of 46 to 52 modules were detected at most of the sparsities (only 22 modules detected in *S* = 0.033%). Each of these modules was assigned a different color, with which most of the brain regions in the classic atlas can be identified. The characteristic areas included the medial prefrontal cortex, the dorsal lateral frontal cortex, the sensory motor area, the supplementary motor area, the dorsal and ventral precuneus, the anterior and posterior inferior parietal lobule, the medial and lateral temporal cortex and temporal pole, the visual cortex, and the anterior and posterior insular.

#### 3) Degree distribution and hubs

**Figure 7:**
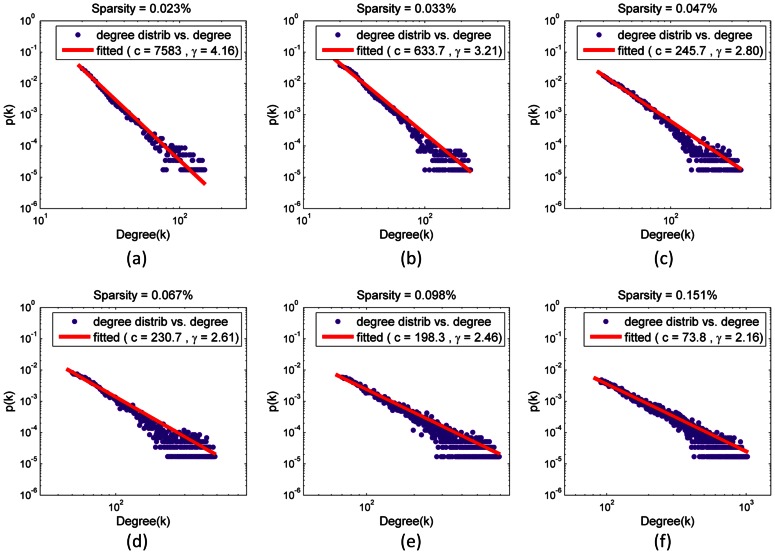
Distribution of the nodal degree under 6 sparsities (log-log plot). Panel (a)∼(f) are the degree distribution and the fitting result of the network with a sparsity of 0.023%, 0.033%, 0.047%, 0.067%, 0.098%, and 0.151%, respectively. The spot lines reflect the probability of finding a node connected to a given number of neighbors; the red solid lines indicate the curve fitted results of the power law 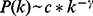. The estimated exponent *γ* under 6 sparsities are 4.16, 3.21, 2.80, 2.61, 2.46 and 2.16, respectively.

**Figure 8:**
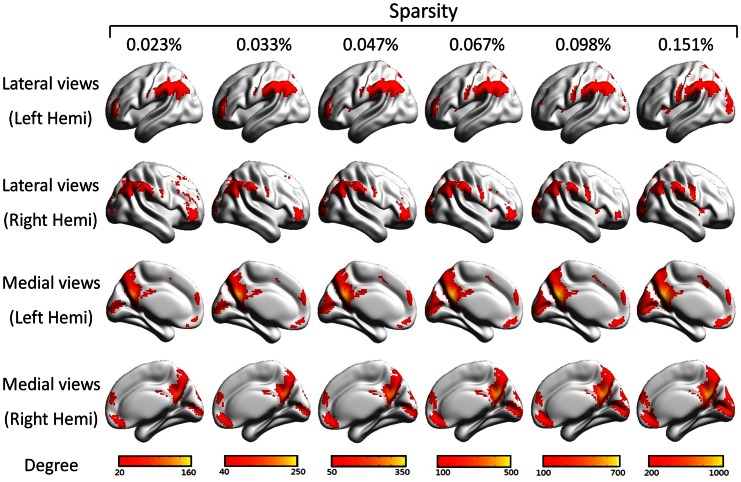
The degree of voxels near the cerebral cortex under different sparsities. Nodes that have a much higher degree than average are considered to be potential hub-voxels. We mark the voxels with degrees that are one standard deviation above the mean. Voxels with a higher degree are in yellow, and voxels with a lower degree are in red. Across different sparsities, some hub-areas are stable, including the bilateral precuneus (PCUN) and posterior cingulate cortex (PCC), the medial prefrontal cortex (MPFC), the lateral prefrontal cortex (LPFC), and the inferior parietal lobule (IPL) (containing the angular gyrus and the supramarginal gyrus). This figure was visualized with the BrainNet Viewer (http://www.nitrc.org/projects/bnv/).

We further examined the degree distribution of voxel-based functional brain networks. The degree distribution of the brain networks (Fig. 7) fitted a power law scaling well, decaying as *p*(k) ∼ *ck* ^−*γ*^ on a log-log plot, with an estimated exponent *γ* ranging from 2.16 (*S* = 0.151%) to 4.16 (*S* = 0.023%). This power law indicated that the voxel-based function brain networks were scale-free, with a small number of brain regions having many connections with most other regions. Aside from the degree distribution, we also pay close attention to those nodes that have a high degree of connectivity, which are often considered to be hub regions [7], [58]. Here, voxels with a degree that is one standard deviation above the average degree are defined as hub-voxels. Several brain regions were identified stably over all of the sparsity thresholds. These regions belonged mainly to the default mode network (DMN), including the bilateral precuneus (PCUN) and posterior cingulate cortex (PCC), the medial prefrontal cortex (MPFC), the lateral prefrontal cortex (LPFC), and the inferior parietal lobule (IPL) (containing the angular gyrus and the supramarginal gyrus). Additionally, we found a higher connecting degree in the medial visual cortex and the sensorimotor cortex. Parts of the bilateral visual cortex were also identified (Fig. 8).

## Discussion

We have presented a CPU-GPU hybrid framework for accelerating the construction and analysis of voxel-based functional brain networks. Taking advantage of the super data-parallel computing capability of GPUs, our hybrid framework greatly reduced the computational time compared to the traditional CPU platform, and finished the prohibitive computation in an acceptable amount of time. With the proposed framework, we analyzed the group brain networks that were constructed from 197 subjects and revealed small-world properties and modular structure in the voxel-based functional brain networks. Furthermore, highly connected hubs were observed in the medial frontal, parietal and occipital cortex regions.

### CPU-GPU Hybrid Framework

Researchers are focusing more attention on brain network analysis. At the same time, the computation budget of brain network analysis is becoming heavier because of the growing quantity of subjects in datasets and the increasing resolution used when constructing brain networks from a coarse to a fine granularity level. Therefore, an efficient, accessible and scalable computational platform is required for brain network analysis.

Our hybrid framework was set up with multi-core CPUs and GPUs. CPUs and GPUs have different design philosophies. A large amount of CPU chip area is dedicated to caching and branch prediction [59]. In contrast, GPUs devote more transistors to data-parallel arithmetic operations and have far more processing elements and a higher memory bandwidth. We accelerated the correlation calculation, the APSP calculation and modular detection with GPUs because these algorithms have a large amount of parallelism and do not require complex flow control which is suitable for using GPU calculations. For these three procedures, we achieved promising speedups (1.6∼2.6x for modular detection, above 100x for the other two) over a single-thread C/C++ implementation on a CPU. It is worth mentioning that CPU vendors provide high-performance math libraries, such as Intel MKL and AMD ACML. Routines in these libraries are highly optimized and can be up to 10 times more efficient than our C/C++ implementation. These routines can assist during some of the brain network analysis procedures, e.g., Pearson’s correlation and modular detection, because they can be expressed as basic linear algebra subprograms (BLAS). With the aid of high performance math libraries, CPUs can achieve similar performance to GPUs in modular detection; however, for Pearson’s correlation, the CPU implementation is still slower than the GPU implementation. Other procedures, such as APSP and the calculation of betweenness, cannot take advantage of the math libraries, and it is extremely difficult for typical developers to achieve the best performance for CPUs (such as the vendor-provided libraries).

Another possible solution to the very large amount of computation required is large-scale clusters. However, large-scale clusters often reside at computing centers and are not easily accessible to most researchers. In contrast, the hardware used in our framework is a personal computer equipped with a GPU as an accelerator. Our framework also has the advantage of having a low cost, a small size and low power consumption, and can be integrated into future MRI machines because of these advantages. At the same time, our framework is also useful if one would like to use clusters for brain network analysis. In the state-of-the-art high-performance clusters, individual nodes often have both CPUs and GPUs (http://www.top500.org/), which is exactly the architecture our framework is intended to target.

In the section on Speedup and Performance results, we have presented a performance comparison between our hybrid framework and a single-thread CPU implementation, involving two different algorithms for the calculation of the characteristic path length (*Lp*) and the modular detection algorithm. It is worth noting that the modular detection and blocked FW algorithms implemented on the GPU were not as fast as we expected because of the low range of sparsities in our experiments (the highest sparsity is 0.151% lower than 2%). As shown in table 1, we can find the gradual trend that the modular detection of networks with a higher sparsity can obtain a higher speedup on a GPU compared to a CPU. Also shown in Fig. 5, the GPU blocked FW algorithm is more suitable for denser networks that have a sparsity higher than 2%. The low range of sparsities in our experiments could be caused by the regression of the global signal and by averaging the correlation coefficient matrix across the subjects. As described in a recent study [60], the removal of the global brain signal rendered the correlation coefficient *r_ij_* (elements from the average correlation matrix of the group of subjects) centered close to zero. Therefore, compared to conditions without any global signal removal and with the same level of threshold *^r *corresponding to the same level of p-value, we would obtain a lower sparsity. In short, the GPU blocked FW algorithm will have a better performance and help substantially when we analyze brain networks that have high sparsities in future work.

Our framework is also scalable to a larger network size. For example, in Pearson’s Correlation and the APSP calculation on a GPU, the network is divided into blocks to allow the handling of arbitrarily large networks as long as there is sufficient memory for the CPU to access. In modular detection, although it is difficult to handle networks by each block during the process, only sparse networks, which require little memory, are stored on the GPU, which ensures the scalability of our platform.

### Voxel-based and Region-based Brain Network Analysis

Voxel-based human brain functional networks were constructed and analyzed in this paper. Compared with ROI-based brain networks, voxel-based brain networks have several advantages. First, recent studies have demonstrated that network properties, such as small-worldness, network efficiency and degree distribution, were influenced by the definition of a node [33], [61]. Constructing brain networks based on voxels overcomes the difficulties of anatomically or functionally defined brain regions. Second, the low resolution of region-based brain networks might lead to the loss of some important connectivity information [34], especially for regions that contain multiple sub-divisions. Many associated cortices involved in multi-functions can be further divided into functionally discrete subdivisions, such as the hippocampus and amygdala region [62], the motor cortex [63], the lateral parietal cortex [64], and the medial parietal cortex [35]. Mixed connectivity information might be involved and lead to confusion when a low resolution atlas is used. Third, voxel-based brain networks provide better spatial localization ability. When a highly connected node is identified, the region-based network can only identify the whole cortex region as a hub; in contrast, a voxel-based network can tell exactly which parts of the cortex serve as hubs [7], [58], [65]. With the above advantages and the progress of computing power, high-resolution brain network analysis will bring a more detailed perspective to human connectome studies.

### Biological Findings

Our results identified the small-world property, modular structure and highly connected hubs in voxel-based functional brain networks during the resting state. The small-world model characterizes the architecture of a network that has both well-connected local neighborhoods (a high clustering-coefficient) and a short topological distance between two long-range nodes (a short characteristic path length). Such a structure is observed in a series of structural and functional connectome studies, in which brain networks were constructed based on regions [6], [13] and based on voxels [34], [58]. These findings indicate that the human brain possesses both local functional specialization and high global communicational integration, which is an optimized organizational pattern of evolution.

Strongly interconnected sub-networks corresponds to the significant modular structure of brain networks. The modular architecture contributes to various aspects of the functional organization of the human brain, such as efficient local information processing within modules [66], [67], the balance of functional segregation and integration, and high resilience to network node or edge damage [12]. We identified several modules combined from brain areas based on voxel-wise functional brain networks using the classic Newman’s spectral method. The segmented brain regions possessed similar but not identical patterns, corresponding to those parcellated cortex areas that are in charge of diverse functions in classical brain anatomy atlases [68], [69]. Recent studies, along with the graph theoretical modularity analysis method, also attempted to learn functional brain organization in a voxel-wise way and demonstrated detailed functional segment results in comparison with ROI-based studies [52], [70]. Voxel-based modularity studies might provide a view of the fine-grained scale in the functional network topological organizations, and might offer precise parcellation over the cortex and subcortex.

The nodal degree is the most common metric that reflects the importance of nodes in terms of direct connections [7]. In our study, we used the nodal degree to measure the importance of each voxel in the functional brain network. We identified several brain regions as network hubs, which mostly belonged to the DMN, including PCUN and PCC, MPFC, LPFC, and IPL. These DMN regions, especially the PCUN, the PCC, and the MPFC, have already been demonstrated to be core regions in studies of metabolism [71], anatomical networks [8], [9], [72], and functional networks [65], [73]. Our finding verified the inference that the DMN regions play a key role in brain function integration by their various communications with other dispersed brain regions.

Our results showed that the degree distribution of voxel-based functional brain networks followed a clear power law scale decay, which is indicative of a scale-free network with the existence of richly connected hubs and is coincident with previous voxel-based functional brain network studies [58], [74], [75]. However, truncated power law degree distributions have also been reported from many other brain network studies performed on region-based network constructions, including both the functional networks [33], [73] and structural networks [8], [11]. The truncated power law networks are less vulnerable to attack than scale-free networks; nevertheless, both networks are robust to random attacks compared to random networks [73]. The reasons for the reports of these two types of network embedded in the human brain network are currently unclear; however, the scale-free degree distributions were reported only in high spatial resolution network studies [58], [74], [75]. Furthermore, a recent voxel-based functional network study [34] reported truncated power law degree distributions under a complimentary cumulative distributions fitting. Therefore, we infer that the network degree distribution might be influenced methodologically by the spatial resolution of the nodal definition and the curve-fitting method.

Furthermore, the above network properties of functional networks in those individuals who have neuropsychiatric disorders often changes [76], [77]. However, because of computational limitations, most of the present studies defined network nodes in the ROI-based level using multifarious atlas or custom-defined ROIs; voxel-based network analyses have rarely been performed. There are still many controversial results in these studies, such as in Alzheimer’s disease [25], [78]. Considering that the ROI-defined brain regions often comprise functional heterogeneous voxels, the voxel-based network analysis could eliminate the potential methodological confounding factor that arises from the various nodal definitions.

### Conclusions

In this work, we propose a hybrid CPU-GPU framework for human connectome studies. Utilizing the computation power of both types of hardware, the whole process for one network finishes much faster than with traditional methods and takes an acceptable amount of time. The main purpose of our work is to stress that the advancement in parallel computing technologies can make revolutionary contributions to neuroscience research. In the future, we will continue to demonstrate our platform and integrate new modules and methods, such as DTI modeling and fiber tractography [79]. We have published our toolbox online at http://www.parabna.weebly.com/. A website is also under construction to provide our results on the 197 subjects’ functional brain networks.
