# Correction: A Hybrid CPU-GPU Accelerated Framework for Fast Mapping of High-Resolution Human Brain Connectome

**DOI:** 10.1371/annotation/b93e8f81-3f0b-41d4-a725-0c54fd99d239

**Published:** 2013-09-10

**Authors:** Yu Wang, Haixiao Du, Mingrui Xia, Ling Ren, Mo Xu, Teng Xie, Gaolang Gong, Ningyi Xu, Huazhong Yang, Yong He

In the Funding Statement, the number of the grant from Microsoft Research Area, National Basic Research Program of China is incorrect. In addition, an additional grant from the Natural Science Foundation of China was incorrectly omitted from the Funding Statement.

The Funding Statement should read: "This work was supported by the Microsoft Research Asia, National Basic Research Program of China (973) (Grant No. 2013CB329000), National Science Fund for Distinguished Young Scholars (Grant No. 81225012), Beijing Natural Science Foundation (Grant No. Z111107067311036) and Natural Science Foundation of China (Grant No. 81030028 and 61171002). The funders had no role in study design, data collection and analysis, decision to publish, or preparation of the manuscript." 

